# Dyslipidemia in Pediatric Patients: A Cross-Sectional Study

**DOI:** 10.3390/medicina59081434

**Published:** 2023-08-08

**Authors:** Andreea Teodora Constantin, Corina Delia, Lucia Maria Tudor, Ioana Rosca, Ana Daniela Irimie, Leonard Năstase, Ioan Gherghina

**Affiliations:** 1 Faculty of Medicine, University of Medicine and Pharmacy “Carol Davila”, 020021 Bucharest, Romania; andreea.constantin@drd.umfcd.ro (A.T.C.);; 2 Pediatrics Department, National Institute for Mother and Child Health “Alessandrescu-Rusescu”, 020395 Bucharest, Romania; corina_delia@yahoo.com (C.D.);; 3 Faculty of Biology, University of Bucharest, 030018 Bucharest, Romania; 4 Faculty of Midwifery and Nursery, University of Medicine and Pharmacy “Carol Davila”, 020021 Bucharest, Romania; 5 Neonatology Department, Clinical Hospital of Obstetrics and Gynecology “Prof. Dr. P.Sârbu”, 060251 Bucharest, Romania; 6 Neonatology Department, National Institute for Mother and Child Health “Alessandrescu-Rusescu”, 020395 Bucharest, Romania

**Keywords:** dyslipidemia, pediatric, lifestyle

## Abstract

There is an increasing interest in dyslipidemia in adult patients since it is known to contribute to early cardiovascular disease. Often, dyslipidemia starts in childhood, and it is associated with aggravating lifestyle choices concerning eating habits, such as the tendency to consume processed food and fast food, as well as the tendency to be more and more sedentary. We conducted a retrospective cross-sectional study describing the prevalence of dyslipidemia in a single medical center in Romania and the associated pathology. We evaluated all lipid profiles that were ordered in our clinic over nine years. We included 2413 patients that were evaluated in our clinic in the timeframe 2011–2020. Out of them, 18.23% had high values for LDL-cholesterol. More than a quarter (25.91%) were diagnosed with obesity. 11.37% of the patients with high LDL-cholesterol levels had various metabolic disorders including primary dyslipidemia. A small number of patients with hypercholesterolemia had thyroid disorders (4.10%). Patients with high LDL-cholesterol had various diagnoses ranging from metabolic to neurologic disorders, keeping in mind that there are multiple pathologies that can lead to dyslipidemia. Evaluating children for dyslipidemia is at hand for medical professionals. Screening for dyslipidemia in children would provide the opportunity to prevent rather than treat cardiovascular events.

## 1. Introduction

Dyslipidemia is defined by high levels of plasma cholesterol and/or triglycerides (TG) or a low level of high-density lipoprotein (HDL) cholesterol. The long-term effect of dyslipidemia is the development of atherosclerosis [1]. The association between childhood risk factors and the development of atherosclerotic cardiovascular disease in adulthood has been recently proven [2,3]. Certain factors, such as hyperlipidemia, hypertriglyceridemia, hypertension, and obesity at a young age, can accelerate atherogenesis [4]. 

There are four classes of lipid disorders in pediatric patients. Other than lifestyle-related dyslipidemia and medication-related dyslipidemia, there are genetic lipid disorders that include familial hypercholesterolemia, familial chylomicronemia syndrome, and familial combined hyperlipidemia [5,6]. Certain pediatric conditions, other than obesity, are also characterized by a high risk of developing atherosclerotic cardiovascular disease. Some examples include premature birth, endocrine disorders, chronic kidney disease, organ transplant recipients, and surviving childhood cancer [4]. 

The National Heart, Lung, and Blood Institute (NHLBI) in the United States of America released in 2011 guidelines that recommend universal screening for lipid disorders in children 9–11 years old and a second time at 17–21 years. Additional focused screening is recommended for children with risk factors at a younger age (over two years old) [6]. The same guidelines recommend that the screening should include dosing fasting total cholesterol (TC), low-density lipoprotein cholesterol (LDL-cholesterol), HDL-cholesterol, triglycerides, or non-fasting total cholesterol and HDL-cholesterol [6]. 

Dietary intervention is the first step in managing dyslipidemia. The recommendations include diets lower in saturated fatty acids that may reduce the LDL-cholesterol level. Often, the result of dietary intervention alone is modest [7]. 

The aim of this study was to identify and describe lipid disorders in pediatric patients in our clinic over a 9-year period (2011–2020).

## 2. Materials and Methods

We conducted a cross-sectional, retrospective descriptive study at National Institute for Mother and Child Health “Alessandrescu-Rusescu” in Bucharest, Romania.

We evaluated all lipid profiles that were ordered in our clinic over nine years (2011–2020). Inclusion criteria were blood work done in our clinic, aged under 18 years old. We did not take into consideration whether the blood work was recommended by a physician in our clinic or not. We do not have information on whether the blood work was drawn after the recommended fasting period or not. 

The institutional medical software used in our clinic is Hipocrate. The patient search was based on the investigation number. The database included over 3000 records for lipid panels. Duplicates (multiple evaluations for the same patient) were excluded from the final database, keeping only one record for each patient. 

We divided the patients into six age groups: 0–2 years old, 3–5 years old, 6–8 years old, 9–11 years old, 12–14 years old, and 15–18 years old. 

Patients with LDL-cholesterol over 130 mg/dL were selected from the database, and using the information available in the system, we searched for additional data about their provenience and diagnosis (according to the International Classification for Diseases (ICD) 10 classification). The value for the LDL-cholesterol level criteria was defined according to the Romanian Pediatrics protocol [8]. Acceptable, borderline, and abnormal levels for total cholesterol, LDL-cholesterol, and triglycerides were defined according to the National Cholesterol Education Program (NCEP) Expert Panel on Cholesterol Levels in Children [9]. 

Biochemical determinations were performed on the separated serum, and they envisaged the next parameters: Total Cholesterol, HDL-Cholesterol, LDL-Cholesterol, and Triglycerides by means of the spectrophotometric (colorimetric) methods as recommended by Roche Diagnostics GmbH, Mannheim, Germany. The reaction products were read by the automatic biochemistry analyzer COBAS INTEGRA 400 PLUS (Roche Diagnostics GmbH, Mannheim, Germany).

Descriptive and statistical analysis was performed using Epi Info™ For Windows, version 7.2. Parametrical or non-parametrical tests used considered the significance level threshold at 0.05.

## 3. Results

### 3.1. Database

The database included 2413 pediatric patients that were evaluated in our clinic in the timeframe 2011–2020. More than half of the patients were female (51.22%). The mean age of the patients was 7.16 years (±5.90). The mean age for girls was similar to the mean age for boys (7.28 years (±6.66) vs. 7.05 years (±4.98)). 

The mean total cholesterol level in the study group was 159.30 mg/dL (±46.52), while LDL-cholesterol mean value was 102.24 mg/dL (±39.21). HDL-cholesterol mean value was 49.83 mg/dL (±18.13). Triglycerides’ mean value was 97.85 mg/dL (±70.42).

The group was divided into age groups. Most of the patients are in the group 0–2 years, 25.40%. The fewest patients are in the group 15–18 years, where there were only 8.66% of the patients.

**Figure 1:**
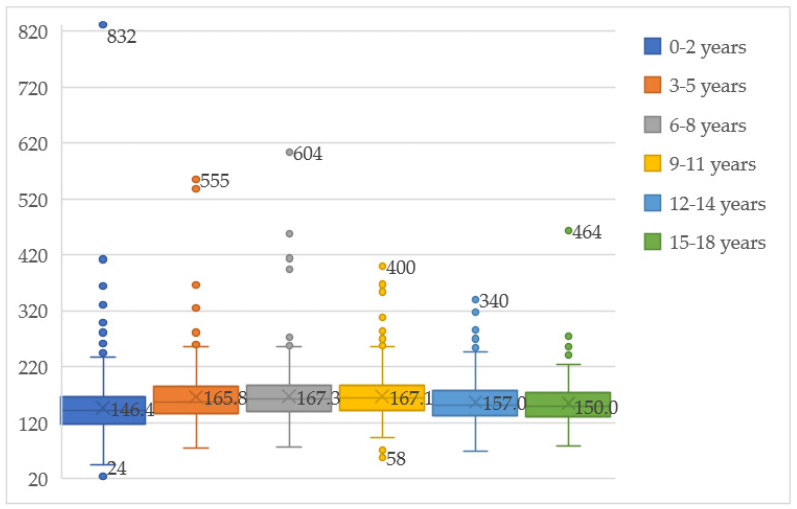
Cholesterol levels by age group. The highest mean cholesterol level was in the age group 6–8 years, while the lowest cholesterol level was in the group 0–2 years.

Total cholesterol levels by age group are represented in Figure 1. The highest mean total cholesterol level was in the age group 6–8 years, 167.32 mg/dL (±44.91). The lowest mean total cholesterol was in the age group 0–2 years, 146.41 mg/dL (±51.99). In every age group, there were patients with abnormally high values. The highest total cholesterol level was in the age group 0–2 years, 832 mg/dL. In all age groups, 11.85% of the children had high total cholesterol levels (≥200 mg/dL), while 21.67% had borderline levels (170–199 mg/dL).

Total cholesterol, LDL-cholesterol, HDL-cholesterol, and triglyceride mean values by age group are presented in Table 1. The highest mean LDL-cholesterol level is in the age group 9–11 years (111.01 mg/dL (±37.24)) and the smallest mean values in the age group 0–2 years (88.26 mg/dL (±37.13)). 

In total, 18.23% of the children had high LDL-cholesterol levels (≥130 mg/dL), while 17.07% had borderline levels (110–129 mg/dL).

The lowest mean HDL-cholesterol was in the age group 0–2 years (41.35 mg/dL (±15.44)) while the highest mean values were in the age group 6–8 years (55.59 mg/dL (±14.18)).

Regarding triglycerides, by far the highest mean values were in the age group 0–2 years (135.41 mg/dL (±101.28)), and the lowest mean values were in the age group 3–5 years (72.61 mg/dL (±36.92)). Out of the patients aged 0–9 years, 13.51% had borderline triglycerides levels (75–99 mg/dL), while 31.82% had high triglycerides levels (≥100 mg/dL), most of the children with high triglycerides levels being in the age group 0–2 years (21.6%). Out of the patients aged 9–18 years, 20.85% had borderline triglycerides levels (90–129 mg/dL), while 31.97% had high triglycerides levels (≥130 mg/dL).

### 3.2. Patients with High LDL-Cholesterol

There were 440 patients (18.23%) with LDL-cholesterol values above 130 mg/dL. The mean age of the patients included in this group was 7.90 years (±4.46). Almost half of the patients were girls (47.50%). Most of the patients (69.09%) came from urban areas, and the majority (44.77%) came from the capital, Bucharest. 

The mean total cholesterol level in the study group was 218.57 mg/dL (±58.46). The highest total cholesterol level was in the age group 0–2 years (233.27 mg/dL (±90.51). Mean LDL-cholesterol reached 161.44 mg/dL (±41.31), while mean HDL-cholesterol was 53.00 (±26.26). The highest LDL-cholesterol mean level was in the age group 3–5 years (167.21 mg/dL (±51.50)), while the lowest HDL-cholesterol was in the age group 0–2 years (47.03 mg/dL (±17.47). 

The diagnostic results of the patients with high LDL-cholesterol were considered (see Table 2). More than a quarter (25.91%) were diagnosed with obesity. 11.37% of the patients with high LDL-cholesterol levels had various metabolic disorders (involving aromatic amino acids metabolism, lactose intolerance, thesaurismosis, and cystic fibrosis), including primary dyslipidemia. Out of them, 80% (40 patients) were diagnosed with dyslipidemia, most of them (28 patients) with pure hypercholesterolemia. A small number of patients with hypercholesterolemia had thyroid disorders (4.10%), most of them being diagnosed with congenital hypothyroidism. In total, 3.21% of the patients had psychiatric disorders, while 2.51% had neurologic disorders.

For a large number of patients (28.65%), data regarding a diagnosis explaining their increased cholesterol level was missing. They are probably patients that came to our clinic for routine bloodwork prescribed by their pediatrician (outside our clinic) or family doctor.

Lipid panel mean values by pathology can be consulted in Table 3. The highest mean values for all components of the lipid panel were recorded in pediatric patients with disease of the genitourinary system, most of them being diagnosed with nephrotic syndrome. 

## 4. Discussion

Dyslipidemia is characterized by high total cholesterol, high LDL-cholesterol, high non-HDL-cholesterol, high triglycerides, and/or low HDL-cholesterol. In the general pediatric population, the prevalence of abnormal lipid values is estimated at around 8–20% [10]. Studies on the prevalence of dyslipidemia in Germany [11] report that 6.1% of children and young adults had LDL-cholesterol ≥ 130 mg/dL. In the United States, estimates on the prevalence of dyslipidemia in children ages 6 to 19 years old are around 20%, with low HDL and elevated TG being the most prevalent abnormalities [12,13]. In Romania, an estimated 67.1% of adults have at least one lipid abnormality [14]. In our study, 18.23% of the patients had high LDL-cholesterol levels (≥130 mg/dL), a result situated towards the higher end. In our country, there is no active screening program for dyslipidemia in children. 

Normal values for cholesterol levels vary by age and sex [6,15]. Although some studies [5,16] say that boys tend to have higher levels of HDL-cholesterol and TG as well as lower LDL-cholesterol than girls, in our study, there was no statistically significant difference between genders. Normally, there is fluctuation in cholesterol levels during childhood and adolescence. In boys, the peak is reached before puberty, at the age of 9–11 years [9]. 

Hypertriglyceridemia can result from reduced TG clearance or increased TG production [17]. Severe hypertriglyceridemia raises concerns in childhood and adolescence because it can cause acute pancreatitis [18]. In pediatric patients, elevated TG levels can be caused by genetic mutations in the genes involved in TG metabolism or by environmental and exogenous conditions [19,20]. Due to reduced protein lipase activity, limited adipose stores, and immaturity, preterm and critically ill children may be particularly prone to hypertriglyceridemia [21]. In our study, 31.82% of the children aged 0–9 years had high triglyceride levels (≥100 mg/dL), and 21.6% of those aged 0–2 years. We can safely assume that most of the children aged 0–2 years are breastfed or receive some kind of milk formula. In this age group that is fed either human milk or formula, triglyceride concentrations are often high (150 mg/dL to 200 mg/dL) [22,23,24]. The very high mean value of triglycerides in the youngest group can be explained by their feeding habits and timing. From a young age, infants are fed at short intervals of time, and the probability that their bloodwork was drawn in a non-fasting state is higher than in older children. The diet of young children and especially infants, is based on milk (being either breastfed or formula-fed). The lipid composition of formula and breastmilk, consisting primarily of TG, is not dissimilar [25]. Infant lipid profiles appear to reflect the lipid composition of the milk intake [26]. In human mature milk, lipid averages 3.5 to 4.5/100 g but changes with the length of time the mother has been breastfeeding, during the course of a day, and increases during individual feeding [27]. In patients with hypertriglyceridemia, nutritional intervention, based on restriction of lipids and simple sugars, is essential [18].

Obesity and the condition of being overweight affect both children and adults and has become a world epidemic [28,29,30,31]. High levels of LDL-cholesterol and triglycerides are present in adolescents with central obesity [32,33,34]. In patients with obesity, adipocyte hypertrophy (in response to increased caloric intake) and adipokine dysregulation lead to sodium retention, activation of the renin-angiotensin-aldosterone system, hypertension, insulin resistance, and increased inflammation [35,36]. The chronic inflammatory state that occurs in obesity causes metabolic complications such as dyslipidemia, hepatosteatosis, and cardiovascular disorder [37]. In our study, more than a quarter of the patients with high LDL cholesterol were diagnosed with obesity (25.91%). In another recent study from Western Romania [38], the prevalence of overweight and obesity in children at school age was estimated at under 30%, an estimation consistent with our findings. The prevalence of overweight and obese children in European countries has significant variations from 9% in Slovakia to 26% in Italy and 29% in the United Kingdom [39]. It is known that obesity increases the risk of developing dyslipidemia. Regardless, almost half of the adolescents with dyslipidemia have normal weight, and there is also a large number of obese adolescents with normal cholesterol levels [5,40]. 

Congenital hypothyroidism is diagnosed in 1 in 2000 to 4000 live births [41]. It is known that thyroid function regulates many metabolic parameters, and it significantly impacts lipoprotein metabolism [42,43,44,45]. Congenital hypothyroidism modifies the metabolic programming promoting dyslipidemia and hyperleptinemia with a change in the thyroid gland’s function [46]. In affected patients, due to a decrease in the activity of the LDL-receptor, decreased catabolism of LDL and intermediate-density lipoprotein results, thus leading to increased total cholesterol and LDL-cholesterol levels [47,48,49]. In our study, 4.10% of the children with high LDL-cholesterol levels had been diagnosed with congenital hypothyroidism. In other studies, among patients with hypothyroidism, 30% had high levels of total cholesterol and LDL-cholesterol, while 90% had dyslipidemia [43,50]. There is an association between thyroid stimulating hormone (TSH), thyroxine (T4) levels, and lipid profile. Normalizing the thyroid profile allows a reduction of cholesterol, TG, and LDL-cholesterol [51].

Concerning metabolic disorders, 11.37% of the patients with high LDL-cholesterol were diagnosed with this kind of pathology. In total, 9.09% were diagnosed with dyslipidemia and 6.36% with hypercholesterolemia specifically. Familial hypercholesterolemia (FH) is a genetic disorder characterized by high LDL-cholesterol levels. The prevalence of heterozygous FH is estimated at 1:200 individuals [52,53]. Although it is not a rare disease, it is underdiagnosed [54]. It leads to an increased risk of developing cardiovascular disease [53,55]. The pathophysiology of FH relies on the decreased function of the LDL-receptor (LDLr) due to a genetic defect. There are multiple possibilities; LDLr might not be synthesized at all, it might not be properly transported from the endoplasmic reticulum to the Golgi apparatus. In other situations, LDLr does not properly bind LDL on the cell surface or does not properly cluster in clathrin-coated pits for receptor endocytosis. Last but not least, sometimes the LDLr is not recycled back into the cell surface [54,55]. Recently, we published a study from our clinic investigating the genetic diagnosis of FH in 20 pediatric patients [56]. In that study, 8 out of 20 patients had genetic confirmation for FH.

In our study, 3.21% of patients presented with psychiatric disorders and high LDL-cholesterol. Patients with psychiatric disorders have a higher risk of premature mortality, mostly due to cardiovascular disease. Psychiatric conditions are characterized by an increased risk of metabolic syndrome presenting with a multitude of risk factors, including dyslipidemia [57,58]. Dyslipidemia is also associated with psychotropic medications [58,59]. Severe metabolic and cardiovascular adverse effects are seen in pediatric patients, especially when multiple antipsychotics or classes of psychotropic medications are prescribed together [60]. 

The nervous system of mammals contains a high amount of cholesterol, 10-fold more than any other organ [61]. About 70% of cholesterol is present in myelin, while the remaining 30% is divided between neurons (10%) and glial cells (20%) [62]. There is more and more evidence suggesting that disturbances in cholesterol metabolism are associated with the development of various neurologic conditions. In our study, 2.51% of patients had high LDL-cholesterol and neurologic disorders, most of them (1.13%) being diagnosed with epilepsy. Antiepileptic drugs negatively impact LDL-cholesterol levels. The most commonly studied drugs were carbamazepine, valproic acid, and phenytoin, all three causing considerable changes in plasma lipid levels in treated patients [63]. Patients treated with antiepileptic drugs are at increased risk of developing a cardiovascular event [64].

In our study, 2.29% of patients with high LDL-cholesterol were diagnosed with congenital malformations, deformation, and chromosomal abnormalities. Most of them were diagnosed with either Prader–Willi Syndrome or Down Syndrome. Prader–Willi syndrome is a rare genetic disorder characterized by muscular hypotonia, dysmorphic features, low lean body mass, mental retardation, behavioral abnormalities, and an insatiable appetite that leads to morbid obesity [65,66]. In these patients, persistently high serum levels of ghrelin (a hormone produced by gastric mucosa which normally stimulates short-term food intake during starvation) play a central role in promoting hyperphagia, increasing appetite, weight gain, and obesity, thus increasing the risk of obesity-related comorbidities such as dyslipidemia [67]. Down Syndrome is the most frequent viable chromosomal abnormality worldwide [68]. Patients with Down Syndrome have characteristic dysmorphic features and many health issues, including endocrine disorders [69]. It is associated with a high risk of chronic diseases such as being overweight, obesity, and dyslipidemia [70,71]. Studies conducted in pediatric populations show higher rates of dyslipidemia in children with Down Syndrome compared with the general population [71,72]. 

Among the complications of nephrotic syndrome is dyslipidemia, increasing the risk of atherosclerosis and thromboembolism [73]. In nephrotic syndrome, due to a combination of increased production and impaired catabolism of LDL and apoB-100, there are marked elevations of serum, total cholesterol, and LDL-cholesterol [74,75]. Lipid values recorded in patients diagnosed with diseases of the genitourinary system were the highest. The mean total cholesterol value was as high as 432 mg/dL (±114.89), and the mean LDL-cholesterol was 325.00 mg/dL (±111.24). These values can be explained by the fact that there were only four patients with nephrotic syndrome (0.92%). Our hospital does not have a Nephrology department, and patients with nephrotic syndrome are usually cared for in other hospitals in our city. The patients with nephrotic syndrome included in our study were admitted at diagnosis.

Some of the patients with high LDL-cholesterol in our study were diagnosed with diseases of the digestive system, including celiac disease. The association of celiac disease with premature atherosclerosis and cardiovascular disease is controversial [76]. A gluten-free diet can determine a higher intake of simple sugars, proteins, and saturated fat and a lower intake of complex carbohydrates and fibers that can contribute to the development of hypertension, dyslipidemia, diabetes mellitus, metabolic syndrome, and hepatic steatosis [77,78,79].

Our study has certain limitations. It was a single-center study. The addition of several other centers from our country would be needed in order to draw conclusions regarding cholesterol levels and the incidence of cholesterol-related disorders in our country. Another limitation would be the lack of medical information available in the medical software in the beginning years of the software being used. 

## 5. Conclusions

Dyslipidemia can be diagnosed since childhood using a common test available worldwide, the lipid profile. Pediatric dyslipidemia contributes to early atherosclerosis and, therefore, to cardiovascular disease. Evaluating children for dyslipidemia through universal screening would be beneficial for future adults that would have the opportunity to prevent rather than treat cardiovascular events. 

There are certain pediatric patients that have a higher risk of developing dyslipidemia due to certain other disorders. Patients with high LDL-cholesterol included in our study had various diagnoses, from hypothyroidism to neurologic and psychiatric disorders. These groups of patients should be monitored closely for dyslipidemia and should receive lifestyle advice as well as medical treatment when needed. 

When evaluating a child with dyslipidemia, we should keep in mind that there are multiple disorders and treatments that interfere with cholesterol metabolism. 

No matter if it is lifestyle or medication-related dyslipidemia, or even genetic dyslipidemia, the increased risk of cardiovascular events should be taken into consideration and addressed as a prevention method.

In our country, there is no active screening program for dyslipidemia, thus making primary dyslipidemia an underdiagnosed disorder.

## Figures and Tables

**Table 1:** Lipid panel mean values for the study group by age.

	Age Group 0–2 Years (*n* = 613)	Age Group 3–5 Years (*n* = 354)	Age Group 6–8 Years (*n* = 444)	Age Group 9–11 Years (*n* = 455)	Age Group 12–14 Years (*n* = 338)	Age Group 15–18 Years (*n* = 209)
Total cholesterol	146.4 mg/dL (±51.9)	165.9 mg/dL (±50.5)	167.3 mg/dL (±44.9)	167.3 mg/dL (±40.2)	156.9 mg/dL (±40.1)	154.5 mg/dL (±39.3)
LDL-cholesterol	88.2 mg/dL (±37.1)	107.0 mg/dL (±43.6)	108.4 mg/dL (±38.5)	111.0 mg/dL (±37.2)	102.6 mg/dL (±36.0)	101.8 mg/dL (±37.0)
HDL-cholesterol	41.3 mg/dL (±15.4)	53.6 mg/dL (±29.0)	55.5 mg/dL (±14.1)	53.1 mg/dL (±14.0)	50.0 mg/dL (±14.5)	49.1 mg/dL (±13.6)
Triglycerides	135.4 mg/dL (±101.2)	72.6 mg/dL (±36.9)	81.9 mg/dL (±55.6)	87.9 mg/dL (±46.3)	95.5 mg/dL (±54.7)	86.1 mg/dL (±49.4)

**Table 2:** Diagnostics of patients with high LDL-cholesterol (≥130 mg/dL).

Diagnostic	Percentage of Patients Diagnosed (*n* = 440)
Endocrine, nutritional and metabolic diseases Overweight, obesity and other hyperalimentation Metabolic disorders (including familial hypercholesterolemia) Malnutrition Disorders of the thyroid gland (hypothyroidism) Diabetes mellitus	52.75% 25.91% 11.37% 11.14% 4.10% 0.23%
Other	28.65%
Diseases of the respiratory system	3.41%
Mental, Behavioral and Neurodevelopmental disorders	3.21%
Diseases of the digestive system	2.96%
Diseases of the nervous system	2.51%
Congenital malformations, deformations and chromosomal abnormalities	2.29%
Diseases of the musculoskeletal system and connective tissue	1.59%
Diseases of the genitourinary system	0.92%
Diseases of the blood and blood-forming organs and certain disorders involving the immune mechanism	0.69%
Diseases of the eye and adnexa	0.68%
Diseases of the circulatory system	0.23%
Neoplasms	0.23%

**Table 3:** Lipid panel mean values by pathology.

Diagnostic	Total Cholesterol (mg/dL)	LDL-Cholesterol (mg/dL)	HDL-Cholesterol (mg/dL)	Triglycerides (mg/dL)
Endocrine, nutritional and metabolic diseases	216.1 (±48.1)	161.8 (±41.2)	51.7 (±15.0)	110.2 (±63.5)
Other	216.7 (±66.2)	155.1 (±30.0)	57.2 (±42.7)	100.3 (±59.5)
Diseases of the respiratory system	206.5 (±38.4)	151.3 (±21.0)	50.7 (±21.1)	128.2 (±64.9)
Mental, behavioral, and neurodevelopmental disorders	229.4 (±46.1)	176.3 (±38.8)	53.7 (±12.3)	101.3 (±58.0)
Diseases of the digestive system	224.0 (±62.3)	166.8 (±47.5)	52.3 (±20.2)	80.2 (±65.1)
Diseases of the nervous system	222.4 (±38.1)	151.9 (±25.0)	49.0 (±14.1)	135.7 (±69.8)
Congenital malformations, deformations, and chromosomal abnormalities	207.7 (±49.5)	158.9 (±35.5)	41.3 (±16.8)	123.7 (±76.0)
Diseases of the musculoskeletal system and connective tissue	211.8 (±56.6)	158.8 (±42.2)	50.0 (±16.7)	75.1 (±20.3)
Diseases of the genitourinary system	432.0 (±114.8)	325.0 (±111.2)	57.7 (±6.8)	253.7 (±287.9)
Diseases of the blood and blood-forming organs and certain disorders involving the immune mechanism	236.0 (±22.0)	183.6 (±39.3)	42.0 (±2.6)	147.3 (±38.0)
Diseases of the eye and adnexa	205.6 (±33.8)	172.0 (±24.8)	48.0 (±6.0)	99.0 (±24.02)
Diseases of the circulatory system	200.0 (NA ^1^)	138.7 (NA ^1^)	60.0 (NA ^1^)	91.0 (NA ^1^)
Neoplasms	205.0 (NA ^1^)	148.0 (NA ^1^)	52.0 (NA ^1^)	185.0 (NA ^1^)

^1^ NA not applicable; among the patients with high LDL-cholesterol, there was only one patient diagnosed with a disease of the circulatory system and one diagnosed with neoplasia.

## Data Availability

Not applicable.
